# Gene Delivery via Octadecylamine-Based Nanoparticles for iPSC Generation from CCD1072-SK Fibroblast Cells

**DOI:** 10.3390/cimb46110747

**Published:** 2024-11-06

**Authors:** Hanife Sevgi Varlı, Meryem Akkurt Yıldırım, Kadriye Kızılbey, Nelisa Türkoğlu

**Affiliations:** 1 Department of Molecular Biology and Genetics, Institute of Science and Technology, Yildiz Technical University, 34220 Istanbul, Türkiye; hsevgivarli@gmail.com (H.S.V.); meryem.akkurt@outlook.com (M.A.Y.); 2 Central Research Laboratory, Yildiz Technical University, 34220 Istanbul, Türkiye; 3 Basic Sciences, Faculty of Engineering and Natural Sciences, Acıbadem Mehmet Ali Aydınlar University, 34752 Istanbul, Türkiye

**Keywords:** nanoparticle-mediated gene delivery, biotechnology, cell culture, reprogramming

## Abstract

This study presents a novel biotechnological approach using octadecylamine-based solid lipid nanoparticles (OCTNPs) for the first-time reprogramming of human CCD1072-SK fibroblast cells into induced pluripotent stem cells (iPSCs). OCTNPs, with an average size of 178.9 nm and a positive zeta potential of 22.8 mV, were synthesized, thoroughly characterized, and utilized as a non-viral vector to efficiently deliver reprogramming factors, achieving a remarkable transfection efficiency of 82.0%. iPSCs were characterized through immunofluorescence, flow cytometry, and RT-qPCR, confirming the expression of key pluripotency markers such as OCT4, SOX2, and KLF4, with alkaline phosphatase activity further validating their pluripotent state. Following this comprehensive characterization, the iPSCs were successfully differentiated into cardiomyocyte-like cells using 5-azacytidine. Our research highlights the innovative application of OCTNPs as a safe and effective alternative to viral vectors, addressing key limitations of iPSC reprogramming. The novel application of OCTNPs for efficient gene delivery demonstrates a powerful tool for advancing stem cell technologies, minimizing risks associated with viral vectors. These findings pave the way for further innovations in biotechnological applications, particularly in tissue engineering and personalized medicine.

## 1. Introduction

Induced pluripotent stem cells (iPSCs) are created by reprogramming adult cells using various gene transfer techniques and specific transcription factors. iPSCs have the remarkable ability to differentiate into all three embryonic germ layers, enabling them to produce a wide range of cell types through controlled and selective culturing methods [1,2]. Moreover, iPSCs can be generated from cells specific to an individual, facilitating the creation of personalized disease models and potential autologous cell-based therapies. The versatility of iPSCs is demonstrated by their ability to differentiate into various cell types, such as neurons [3], cardiomyocytes [4], and hepatocytes [5]. This capacity for differentiation has significant implications for disease modeling, drug screening, and applications in regenerative medicine.

Traditionally, viral vectors have been extensively preferred for gene transfer due to their high transduction efficiency and ability to achieve long-term gene expression [6]. Nonetheless, concerns about safety, immunogenicity, and limited cargo capacity have spurred the development of non-viral vectors, including liposomes, polymeric nanoparticles, and CRISPR-Cas9-based systems [7]. Non-viral vectors provide benefits such as lower immunogenicity and greater flexibility in cargo size [8]. Recent advancements in iPSC generation methods have aimed at improving efficiency and safety, with emerging approaches such as the use of solid lipid nanoparticles showing significant potential [1,9,10]. In this study, octadecylamine-based solid lipid nanoparticles (OCTNPs) were developed and applied as a new method for gene delivery, being successfully used to reprogram human fibroblast cells.

The controlled modulation of somatic cell reprogramming to avoid unintended pluripotent states is a crucial aspect of regenerative medicine. Using non-viral carriers to deliver reprogramming factors enables the more precise and controlled induction of pluripotency in somatic cells. This strategy reduces the risk of uncontrolled cellular transformations and improves the safety profiles of iPSCs for potential future applications [11]. One essential aspect of iPSC characterization involves assessing pluripotency markers, including SOX2, OCT4, LIN28, and NANOG, through methods such as flow cytometry, immunocytochemistry, and real-time PCR (quantitative PCR) [1,10]. Alkaline phosphatase (ALP) is crucial for characterizing iPSCs and is regarded as a key indicator of pluripotency [12,13].

In addition to reprogramming human fibroblast cells into iPSCs, this study investigates the differentiation of these iPSCs into cardiomyocytes using 5-azacytidine (5-Aza). 5-azacytidine, a cytidine analog and DNA methyltransferase inhibitor, plays a crucial role in promoting cardiomyocyte differentiation. By disrupting DNA methylation patterns, 5-aza effectively stimulates the activation of cardiac-specific genes and signaling pathways essential for cardiomyogenesis [14,15]. The use of 5-aza in cardiomyocyte differentiation presents a promising approach for both research and clinical applications. Characterizing cardiomyocyte-like cells derived from induced pluripotent stem cells (iPSC-CMs) is essential for evaluating their maturity and function. Studies by Farag, Ahmed, et al. (2024) comparing bone marrow-derived and adipose-derived mesenchymal stem cells (MSCs) used 5-azacytidine to promote cardiomyogenic differentiation. The cells were treated with 10 μM 5-aza alone or in combination with growth factors. Both treatments resulted in successful differentiation [16]. Shams, S., M. Naseer, and H. Hassan (2017) et al.’s research on the combined effects of 5-azacytidine and TGF-β in differentiating MSCs into cardiomyocytes demonstrated that this combination improves differentiation efficiency and reduces cytotoxic effects compared to 5-aza alone [17].

GATA-4, a zinc finger transcription factor, is crucial for cardiac development and is recognized as a specific marker for cardiomyocyte differentiation [18].

In this study, the conversion of human fibroblast cells into iPSCs was explored using innovative solid lipid nanoparticles (OCTNPs) as a method for gene delivery. The synthesized lipid-based nanoparticles, which are positively charged, were successfully used and showed no toxic effects on the target CCD1072-SK cells. In addition to the standard reprogramming factors, a plasmid coding for a green fluorescent protein (GFP) marker gene was used to monitor transfection efficiency. The iPSCs generated through this method were characterized and then differentiated into cardiomyocyte-like cells using 5-azacytidine. While cardiomyocyte differentiation using 5-azacytidine has been documented for mesenchymal and embryonic stem cells [19,20], this study represents the first attempt to differentiate iPSCs derived from CCD1072-SK cells into cardiomyocyte-like cells using 5-azacytidine.

## 2. Materials and Methods

The CCD1072-SK human fibroblast cell line was sourced from the cell stocks at Yildiz Technical University’s Molecular Biology and Genetics Laboratory. These are not primary human cells. For this study, the following reagents were procured: DMEM (high glucose, with glutamine), F-12 Nutrient Mixture (Ham’s), Fetal Bovine Serum, Trypsin-EDTA Solution C (0.05%) with EDTA (0.02%) from Biological Industries (Beit-Haemek, Israel), Matrigel basement membrane from Corning (Corning, NY, USA), mTeSR™1 feeder-free maintenance medium, and ReLeSR™ enzyme-free human pluripotent stem cell selection. The plasmid used to transfect CCD1072-SK human fibroblast cells was the Human 4-in-1 iPSC PiggyBac Vector from System Biosciences (Palo Alto, CA, USA). Additionally, Minimum Essential Medium, non-essential amino acids, basic fibroblast growth factor (bFGF), and beta-mercaptoethanol (2-mercaptoethanol) were obtained from Gibco, Life Technologies, Grand Island, NY, USA. IPSC colonies were cultured on a Matrigel matrix from Corning (USA). For iPSC characterization studies, the following antibodies were used for immunostaining: Anti-Oct4 antibody (ab19857), Goat Anti-Rabbit IgG H&L (Alexa Fluor^®^ 488) (ab150077), Anti-SSEA4 antibody [MC813-70] (ab16287), and Goat Anti-Mouse IgG H&L (Alexa Fluor^®^ 568) (ab175473). The ALP Activity Assay Kit (E-BC-K091-M) was obtained from Elabscience, located in Wuhan, China. The cardiomyocyte differentiation agent 5-azacytidine (320-67-2) was purchased from Sigma (Virginia Beach, VA, USA). For cardiomyocyte characterization, the Human GATA4 (GATA Binding Protein 4) ELISA Kit from Elabscience (E-EL-H0882), located in Wuhan, China, was used.

### 2.1. Nanoparticle Synthesis and Characterization

Lipid-based cationic nanoparticles were synthesized for use as gene delivery vectors, incorporating octadecylamine (an 18-carbon fatty amine) as a crucial component. The nanoparticle solution was composed of two phases: a lipid phase containing octadecylamine and chloroform and a surfactant phase consisting of PBS and Tween-80. The emulsion-solvent evaporation technique was used to create nanoparticles. Octadecylamine (0.2 M) was dissolved in chloroform (2 mL) to prepare the lipid solution. The lipid solution was gradually introduced dropwise into an aqueous solution containing 6% Tween 80, and a single emulsion was formed by homogenization with a sonic probe set at 30% amplitude for 50 s. The organic solvent was removed by evaporation under reduced pressure. Nanoparticles were isolated using ultracentrifugation at 6500 rpm at 12 °C for 30 min. The synthesized nanoparticles were dried using a freeze-dryer, and their concentrations were determined. The surface charges and particle sizes of octadecylamine solid lipid nanoparticles (OCT NPs) were determined using a Zetasizer instrument (Malvern 3000 HSA, Worcestershire, UK). The morphology of the OCT NP was examined using FE-SEM (Thermo Scientific Apreo-2S LoVac, Waltham, MA, USA), while the chemical bonding was analyzed using FT-IR (Perkin Elmer, Spectrum 100, Jersey City, NJ, USA).

To establish the non-toxic concentration of the synthesized nanoparticles, an MTT assay was conducted using the CCD1072-SK human fibroblast cell line [21]. The cells were cultured in DMEM + F12 medium supplemented with 10% Fetal Bovine Serum (FBS) and 1% penicillin–streptomycin and incubated at 37 °C in a 5% CO_2_ humidified atmosphere. Cells that reached sufficient density were detached using Trypsin-EDTA, and 10,000 cells were seeded (*n* = 5) into each well of a 96-well plate containing fresh medium. The day after different volumes of OCT NP (5, 10, 15, 20, and 25 μL per well) were introduced into the medium, the samples were incubated for 24 h. After incubation, the medium was removed, and each well was treated with a solution of methyl thiazolyl diphenyl tetrazolium bromide (5 mg MTT/mL in medium), followed by an additional 4 h of incubation at 37 °C with 5% CO_2_. After incubation, the formazan crystals were dissolved in 100 μL of DMSO per well, replacing the MTT solution. The optical density was then measured at 570 nm using an ELISA reader (Thermo Scientific Multiskan Go, Waltham, MA, USA).

### 2.2. Observing Gene Delivery and Transfection Efficiency Using GFP Expression

In the cell culture procedure, a vial of CCD-1072-SK cells (Human fibroblast cell line) was taken from a −80 °C freezer. The frozen vial was quickly thawed in a 37 °C water bath until a small ice crystal remained. In a sterile environment, the contents of the vial were aseptically transferred into a centrifuge tube containing pre-warmed Dulbecco’s Modified Eagle Medium (DMEM) supplemented with glucose and 10% FBS. After this step, the tube was centrifuged at a low speed of 200× *g* for 5 min to pellet the cells. After discarding the supernatant, the cell pellet was resuspended in fresh, pre-warmed DMEM supplemented with 10% FBS and 1% penicillin–streptomycin and transferred to a tissue culture flask. The cells were cultured at 37 °C in an atmosphere containing 5% CO_2_ until they reached 70–80% confluence.

The Human 4-in-1 iPSC PiggyBac Vector containing Oct4-Sox2-Klf4-Myc-GFP plasmid DNA (pDNA) was propagated in *E. coli* JM109 competent cells and purified using a Qiagen Maxi kit (Qiagen, Germantown, MD, USA). DNA quantification was carried out using a Thermo Scientific microDrop plate at 260 nm and 280 nm wavelengths. Then, 10 μL of OCT NP solution (0.5 μg/μL) was combined with 10 μL of plasmid DNA (pDNA) solution (0.2 μg/μL pDNA) to form OCT NP/pDNA conjugates. The solution was then made up to 1 milliliter with distilled water. The zeta potential and size of the OCTNP-pDNA conjugates were determined using a Zetasizer instrument (Malvern 3000 HSA, UK).

A culture medium consisting of DMEM, supplemented with 1% Pen-Strep and 10% FBS, was used for the transfection. In 6-well plates, cells were seeded at a density of 200,000 cells per well and incubated overnight. Subsequently, the culture medium was replaced with serum-free and antibiotic-free DMEM, and the cells were incubated for 1 h to prepare them for transfection.

To prepare the OCT NP/pDNA complex, 10 µL of pDNA solution (0.2 µg/µL) was diluted in 500 µL of serum-free DMEM, followed by the addition of 10 µL of OCT NP solution (0.5 µg/µL). The solutions were then mixed. OCTNPs were mixed with diluted pDNA and incubated for an additional 15–20 min at room temperature to allow for complex formation. Subsequently, 500 μL of the conjugate solution was added to each well, followed by further incubation for 4 h. Following transfection, the culture medium was replaced with DMEM supplemented with 10% FBS, and the plate was then incubated at 37 °C in a 5% CO_2_ atmosphere for 24 h.

After the incubation period, the transfected cells were examined using an inverted fluorescence microscope (Zeiss Axio Observer.Z1, ZEN 2.0) equipped with GFP filters (excitation: 488 nm, emission: 509 nm). Digital images of the GFP-expressing cells were captured, and the transfection efficiency was evaluated by calculating the percentage of GFP-positive cells in relation to the total cell population across multiple fields of view. Transfection experiments were performed in triplicate to ensure the accuracy of the results, using non-transfected cells as controls [22].

### 2.3. Culturing IPS Cells on Matrigel

Matrigel is a gelatinous protein mixture derived from the Engelbreth–Holm–Swarm mouse sarcoma, and it has become an indispensable component in various cell culture and tissue engineering applications. Matrigel comprises laminin, collagen IV, entactin, and growth factors, making it an excellent substrate for supporting the growth of various cell types, including stem cells and primary cells [23,24].

Matrigel was thawed at 4 °C overnight and then coated onto cell culture plates according to the manufacturer’s instructions. The plates were incubated at 37 °C for 1 h to allow the polymers to form. Fibroblast cells that were transfected were selectively harvested from the culture plate using ReLeSR and then seeded into wells coated with Matrigel. The cells were cultured in a 5% CO2 environment, with the culture medium refreshed every 2 days. Morphological changes in the cells on the Matrigel surface were observed using an inverted microscope (Zeiss Axio Observer.Z1) until colony formation was evident.

### 2.4. Characterization of IPS Cells

#### 2.4.1. Assessment of Colony Formation and GFP Expression

Characterizing induced pluripotent stem cells (iPSCs) includes evaluating their colony-forming capabilities and tracking the expression of specific markers, such as GFP, to verify successful reprogramming and pluripotency [25]. Colony formation is commonly used to assess the reprogramming efficiency of induced pluripotent stem cells (iPSCs) derived from somatic cells and their proliferative capacities, which can be observed using an inverted microscope (Zeiss Axio Observer. Z1). The GFP marker gene was carried by the plasmid used to reprogram the cells, enabling expression monitoring using a fluorescent inverted microscope. Monitoring GFP expression offers valuable insights into the efficiency of reprogramming and the sustained maintenance of pluripotency.

#### 2.4.2. Flow Cytometry

The characterization of induced pluripotent stem cells (iPSCs) using OCT-4 and SSEA-4 antibodies through flow cytometry enables the precise quantification of specific markers on the cells of interest, facilitating a comprehensive assessment of the cell population [10,26]. In this study, iPSCs were detached from culture wells using a cell scraper and then subjected to high-speed centrifugation. The obtained pellet was resuspended in ice-cold PBS containing 10% FBS and 1% sodium azide. After aliquoting into Eppendorf tubes, primary antibodies at a concentration of 1 µg/mL were added to the cells, which were then incubated at room temperature for 30 min. After centrifugation, the pellet was resuspended in ice-cold PBS, and fluorochrome-conjugated secondary antibodies were added. After incubation at 4 °C in the dark, unbound secondary antibodies were removed by centrifugation. The labeled cells were resuspended for analysis by flow cytometry. Primary antibodies Anti-Oct4 (ab19857) and Anti-SSEA4 [MC813-70] (ab16287) were used at a dilution of 1:100, and they were paired with the secondary antibodies Goat Anti-Rabbit IgG H&L (Alexa Fluor^®^ 488) (ab150077) and Goat Anti-Mouse IgG H&L (Alexa Fluor^®^ 568), respectively. Flow cytometry was used to quantify OCT-4 and SSEA-4 expression, confirming the maintenance of iPSC pluripotency.

#### 2.4.3. Immunostaining

The iPSCs were characterized through immunostaining using SSEA-4 and OCT-4 antibodies. The primary OCT-4 antibody used was Anti-Oct4 antibody (ab19857), and the secondary antibody employed was Goat Anti-Rabbit IgG H&L (Alexa Fluor^®^ 488) (ab150077). Similarly, the Anti-SSEA4 primary antibody [MC813-70] (ab16287) was used, with Goat Anti-Mouse IgG H&L (Alexa Fluor^®^ 568) being the corresponding secondary antibody. Initially, iPSCs were seeded into 6-well plates and incubated for the appropriate period. They were then separately incubated with SSEA-4 and OCT-4 primary antibodies for 1 h at 37 °C, targeting pluripotency markers [27,28]. After incubation with the primary antibody, iPSCs were washed thoroughly with PBS to remove unbound antibodies. Subsequently, cells were incubated with fluorescently labeled secondary antibodies for 1 h in the dark, followed by a PBS wash to remove unbound antibodies. The results were examined and analyzed using an inverted fluorescence microscope.

#### 2.4.4. Alkaline Phosphatase Activity

The activity of alkaline phosphatase was measured using the Elabscience ALP ELISA kit. The iPSC cell culture medium was replaced with PBS, and cells were subsequently lysed by freezing at −80 degrees Celsius overnight. The standard solution from the kit was diluted, and varying concentrations were added to the control wells, while cell samples were placed in the experimental wells. A working solution was added, and the mixture was incubated before introducing reagent 3. Optical density (OD) values were recorded at 520 nm using a microplate reader, and the results were calculated using the following formula:

$$ALP\;Activity=\frac{\Delta A-b}{a}\times 100$$

ΔA: Absolute OD = (OD_Sample_−OD_Blank_);

*b*: The intercept of the standard curve;

a: The slope of the standard curve.

#### 2.4.5. Quantitative Analysis of Gene Expression for Reprogramming Factors

The gene expression levels of OCT-4, SOX-2, and KLF-4 were analyzed using the RT-qPCR method. RNA was extracted using the GeneJET RNA Purification Kit (Thermo Scientific) following the manufacturer’s protocol. For cDNA synthesis, 1 μL of RNA was reverse-transcribed using the First Strand cDNA Synthesis Kit (Thermo Scientific) according to the provided instructions. Real-time PCR was performed using the AriaMx Real-time PCR System (Agilent Technologies, Santa Clara, CA, USA) with SYBR Green-based Brilliant III Ultra-Fast RT-qPCR Master Mix. Gene expression levels of OCT-4, SOX-2, and KLF-4 were measured in two replicate samples. GAPDH was used as the reference gene, and non-transfected cells (NTCs) served as negative controls.

### 2.5. Cardiomyocyte Differentiation and Characterization

The application of 5-azacytidine (5-aza) is a well-established method in stem cell research for promoting the differentiation of pluripotent stem cells into cardiomyocytes. In the context of cardiomyocyte differentiation, 5-azacytidine (5-aza) has been shown to promote the activation of cardiac-specific genes, aiding the differentiation of stem cells into cardiac lineage [29,30,31]. However, 5-aza can exhibit cytotoxic effects, particularly at higher concentrations. In this study, the MTT assay was used after treating cells with 5-aza for 2 days to assess its cytotoxic effects on iPSCs. iPSCs were seeded onto Matrigel-coated culture plates at 70–80% confluence and allowed to reach full confluence before starting differentiation. The cells were cultured in a medium supplemented with 5-azacytidine (5-aza) at concentrations of 1 µM, 5 µM, and 10 µM for 2 days. At this point, a cytotoxicity analysis was conducted using MTT assays to determine the non-toxic concentration of 5-aza. Once the non-toxic concentration was established, cells were cultured at this concentration for the initial two days, followed by incubation in normal medium for a period of 1 to 2 weeks. The differentiation process was assessed by observing morphological changes with an inverted microscope (Zeiss Axio Observer.Z1).

A key indicator for characterizing induced pluripotent stem cell-derived cardiomyocytes (iPSC-CMs) is GATA-4, a transcription factor known to play a crucial role in cardiac development and function [32,33,34]. The method for characterizing iPSC-CMs includes quantifying specific cardiac markers such as GATA-4 using the Elanscience Human GATA Binding Protein-4 ELISA kit. In this study, to prepare the iPSC-CM samples, cells were lysed to extract protein lysates. The cells were frozen at −80 degrees Celsius overnight to facilitate cell lysis and the release of proteins into the solution. This kit utilized the Sandwich-ELISA technique. The steps were carried out as per the instructions in the user manual. The OD was then determined by measuring the absorbance spectrophotometrically at a wavelength of 450 nm. The results were compared with GATA-4 levels in iPSC-CM samples and normal fibroblast cells to provide insights into their cardiac differentiation and maturation.

## 3. Results

### 3.1. Synthesis and Characterization of OCTNPs

The synthesis and characterization of octadecylamine-based nanoparticles (OCTNPs) were performed to evaluate their physicochemical properties.

The particle size, zeta potential, and polydispersity index (PDI) of the OCTNPs were measured using dynamic light scattering (DLS) with a Zetasizer Nano ZS (Malvern Instruments, Malvern, Worcestershire, UK). The OCTNPs had an average size of 178.9 (±72.71) nm and a narrow size distribution (PDI: 0.1), suggesting a uniform and well-defined nanoparticle population. Importantly, the zeta potential of the OCTNPs was measured at 22.8 (±8.20) mV, indicating a stable colloidal dispersion with a positively charged surface. This positive zeta potential is advantageous for gene delivery applications, as it enhances the interaction of nanoparticles with negatively charged cell membranes, aiding the cellular uptake and intracellular delivery of genetic material [35].

**Figure 1:**
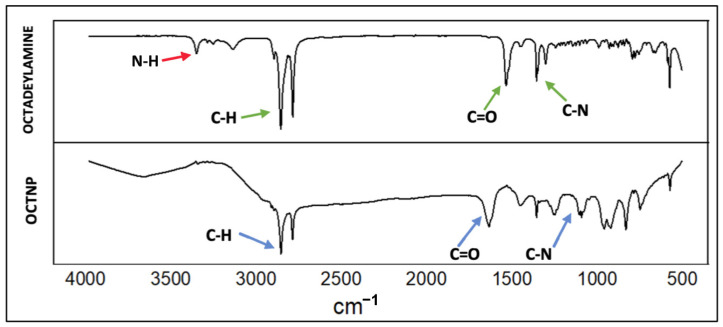
FT-IR spectrum of OCTNP and pure octadecylamine.

A Fourier-transform infrared (FT-IR) analysis was performed to verify the presence of functional groups on the nanoparticle surface, confirming the successful attachment of octadecylamine. The FT-IR spectrum is shown in Figure 1. Pure octadecylamine and octadecylamine incorporated into lipid nanoparticles (OCTNPs) show distinct differences when analyzed through FTIR spectra, revealing insights into how octadecylamine interacts within the nanoparticle structure. Octadecylamine alone has characteristic peaks, like the N-H stretching vibration seen at around 3300–3500 cm⁻^1^. In OCTNPs, if this peak shifts, becomes broader, or less intense, this suggests hydrogen bonding between octadecylamine and the lipids in the nanoparticle. Other important peaks, such as those for C-H stretching around 2800–3000 cm⁻^1^, may also shift in OCTNPs due to changes in octadecylamine’s environment, where it is surrounded by lipid molecules. Additional signals like N-H bending around 1550–1650 cm⁻^1^ and C-N stretching at 1200–1350 cm⁻^1^ may change in the OCTNP spectrum, which would suggest interactions between the amine group of octadecylamine and the lipid molecules, possibly through weak hydrogen bonds or other interactions with lipid head groups. The overall FTIR spectrum of OCTNPs might show broader peaks or slight shifts compared to pure octadecylamine due to the more complex molecular environment within the nanoparticles. In short, the FTIR comparison between pure octadecylamine and OCTNPs can reveal how octadecylamine is incorporated into and interacts within the lipid matrix, particularly through hydrogen bonds or other minor shifts in characteristic peaks. These changes help confirm that octadecylamine is successfully embedded in the lipid nanoparticles. In this study, a comparison of the synthesized nanoparticle with standard octadecylamine showed overlapping peaks, consistent with values reported in the literature for octadecylamine. This alignment confirms the successful integration of octadecylamine into the lipid nanoparticles, validating the effectiveness of the synthesis process [36].

**Figure 2:**
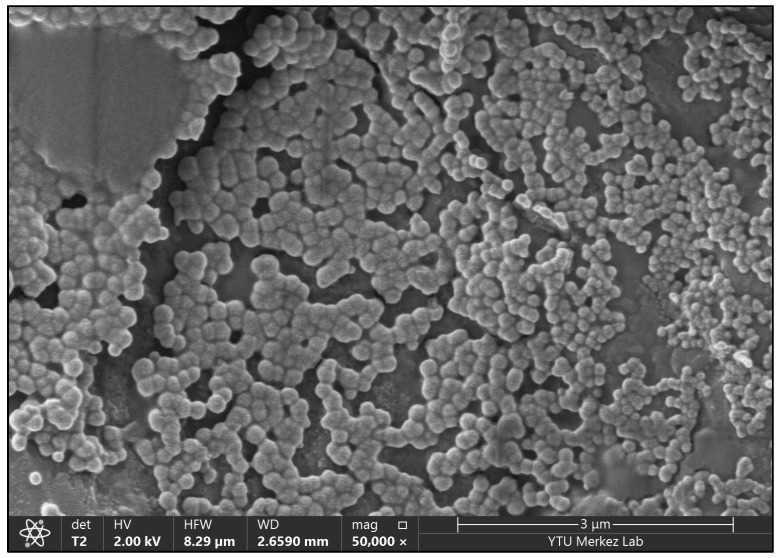
FESEM image of OCTNP.

The morphology and structure of the OCT nanoparticles were analyzed using the Thermo Scientific Apreo 2S LoVac Field Emission Scanning Electron Microscope (FESEM). The particles were coated with Au/Pd and prepared for imaging analysis. The images shown in Figure 2 reveal that the nanoparticles are spherical, and their sizes are consistent with the measurements obtained from the Zetasizer. In the images obtained, the nanoparticles appear round, and their sizes are consistent with the measurements taken using the FESEM.

### 3.2. Cytotoxicity Assessment of OCTNPs

To evaluate the cytocompatibility of OCTNPs, an MTT assay was performed, showing no significant cytotoxic effects on the CCD1072-SK fibroblast cells.

**Figure 3:**
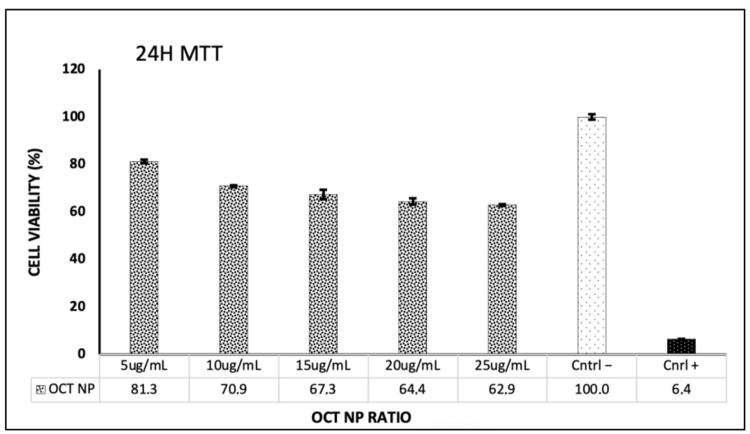
Cytotoxicity analysis of OCTNP.

The cytotoxicity analysis, as shown in the 24 h MTT assay results, indicates a dose-dependent decrease in cell viability with increasing concentrations of OCTNPs. At the lowest concentration tested, 5 µg/mL, cell viability remained relatively high at 81.3%. As the concentration of OCTNP increased to 10 µg/mL, 15 µg/mL, 20 µg/mL, and 25 µg/mL, cell viability correspondingly decreased to 70.9%, 67.3%, 64.4%, and 62.9%, respectively. The culture medium, serving as a negative control, maintained 100% cell viability, thereby confirming the baseline viability when OCTNP was absent. In contrast, the positive control, DMSO, resulted in a significantly reduced cell viability of 6.4%, demonstrating the assay’s ability to detect cytotoxic effects. The results are presented in Figure 3 indicate that the OCTNPs are biocompatible and thus promising candidates for various biomedical applications, particularly in gene delivery.

### 3.3. Monitoring Transfection Efficiency Using GFP Expression

**Figure 4:**
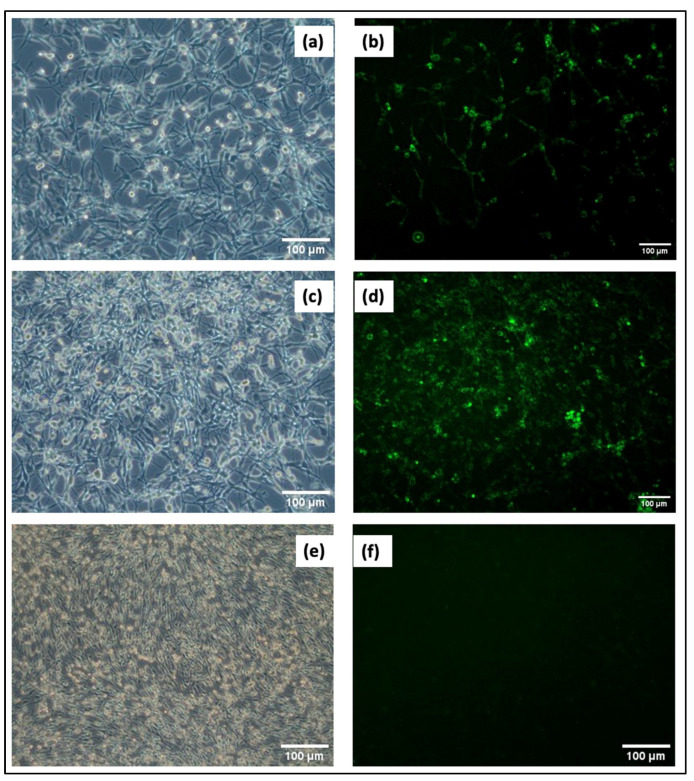
CCD1072-SK transfection images: (**a**) 24 h 10× phase contrast; (**b**) 24 h 10× fluorescent; (**c**) 48 h 10× phase contrast; (**d**) 48 h 10× fluorescent; (**e**) control group (without DNA) 10× phase contrast; (**f**) control group (without DNA) 10× fluorescent.

Nanoparticles interacting with plasmid DNA are likely to increase in size after interaction and exhibit a reduction in zeta potential due to the negative charge of the DNA. The zeta potential and size of the conjugates were measured using a Zetasizer, with each measurement performed in triplicate. The results showed that the conjugates had a size of 874.3 (±62.02) nm and a zeta potential of 3.88 (±3.33) mV, with a PDI of 0.2. Transfection studies were conducted using the obtained conjugates, and images were captured using an inverted microscope at 24 and 48 h. Images from transfections performed without plasmid served as controls. The results are shown in Figure 4. Transfection efficiency was determined to be 82.0% (±6.2) by counting the GFP-expressing cells in three independent regions of the acquired images. Our approach not only showed superior efficacy but also an enhanced safety profile, which is essential for advancing gene delivery technologies. This increased efficiency is especially significant when compared to traditional viral vectors, which are known for their potential cytotoxic and immunogenic effects.

### 3.4. IPSCs Culture on Matrigel

#### 3.4.1. Colony Formation and GFP Expression Assessment

**Figure 5:**
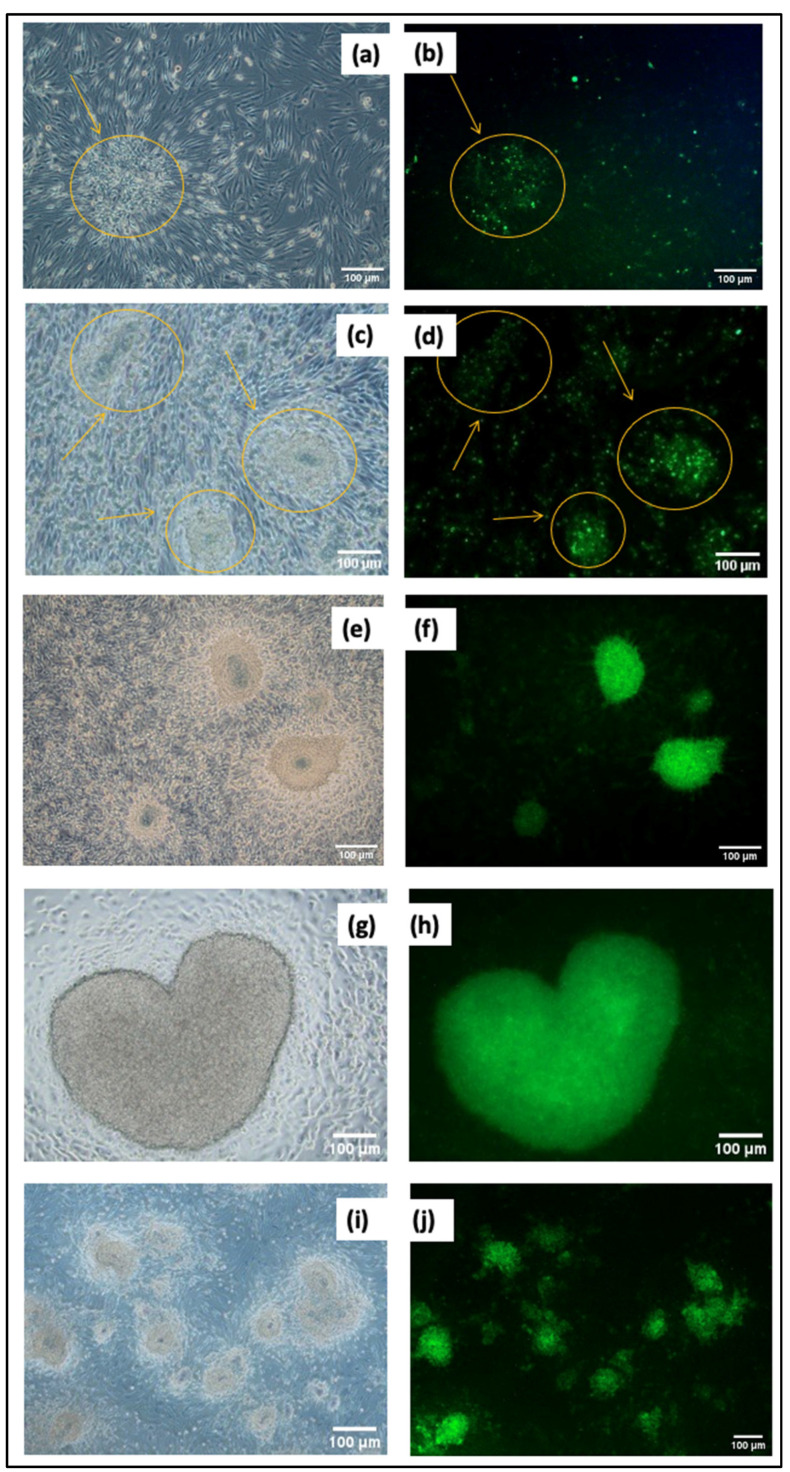
Monitoring colony formation and GFP expression of transfected cells cultured on Matrigel: (**a**) 1st day, 10× phase contrast; (**b**) 1st day, 10× fluorescent; (**c**) 3rd day, 10× phase contrast; (**d**) 3rd day 10×, fluorescent; (**e**) 10th day 10×, phase contrast; (**f**) 10th day, 10× fluorescent; (**g**) 15th day, 20× phase contrast; (**h**) 15th day, 20× fluorescent; (**i**) 20th day, 10× phase contrast; (**j**) 20th day, 10× fluorescent.

Induced pluripotent stem cells (iPSCs) display unique colony morphology when cultured on Matrigel, a commonly used extracellular matrix. iPSC colonies typically have well-defined, compact structures, similar to those of embryonic stem cells. This characteristic morphology indicates that the iPSCs are undifferentiated and pluripotent. The use of Matrigel as a substrate offers crucial signals for cell attachment and proliferation, facilitating the maintenance of iPSC colonies. The accurate monitoring of the iPSC colony morphology on Matrigel is essential for evaluating the quality and pluripotency of the cells during culture and differentiation stages. The plasmid DNA used for gene transfer carries the GFP gene, which is expressed continuously in cells along with reprogramming factors. Monitoring both colony morphology and GFP expression is crucial for the characterization of iPSCs. Figure 5 illustrates the colony-forming behavior of transfected cells cultured on Matrigel over a period of 20 days. A noticeable trend in colonization is observed starting from day 10.

Figure 5a,b illustrate the initial morphology of the transfected cells on the first day of Matrigel culture, as observed under 10× phase contrast and 10× fluorescence microscopy, respectively. The cells appear to aggregate, as highlighted by the yellow circles. The cells continue to cluster and form early colonies, which are visible under both phase contrast (Figure 5c) and fluorescence (Figure 5d) microscopy by the third day of culture. The yellow circles and arrows indicate the developing colonies. The colonies show significant growth by the 10th day of culture, forming more defined and compact structures, which are more pronounced under phase contrast microscopy (Figure 5e) and fluorescence microscopy (Figure 5f). GFP expression confirms that the transfection is successful. On the 15th day of culture, the colonies mature into well-defined structures, including a heart-shaped colony, visible under both phase contrast (Figure 5g) and fluorescent (Figure 5h) microscopy. The images reveal a progressive increase in the number of colonies by the 20th day of culture, as observed under phase contrast in Figure 5i and confirmed by GFP expression in fluorescent microscopy in Figure 5j.

#### 3.4.2. Characterization of iPSCs Using Immunofluorescence Staining

The immunostaining analysis showed distinct fluorescence patterns indicating the expression of pluripotency markers. The iPSC colonies displayed strong green fluorescence, indicating the successful labeling of OCT-4 with Alexa Fluor 488. At the same time, the red fluorescence seen in the same colonies confirmed successful labeling of SSEA-4 with Alexa Fluor 568. These findings indicate that the cells cultured on Matrigel maintained pluripotent characteristics, expressing key markers associated with undifferentiated stem cells. In the characterization of induced pluripotent stem cells (iPSCs), immunostaining with specific markers is essential to confirm pluripotency. Immunostaining was performed using antibodies against OCT-4 and SSEA-4, two well-established markers for undifferentiated pluripotent cells. OCT-4, a crucial transcription factor, and SSEA-4, a surface marker, were stained with Alexa Fluor 488 and Alexa Fluor 568, respectively.

**Figure 6:**
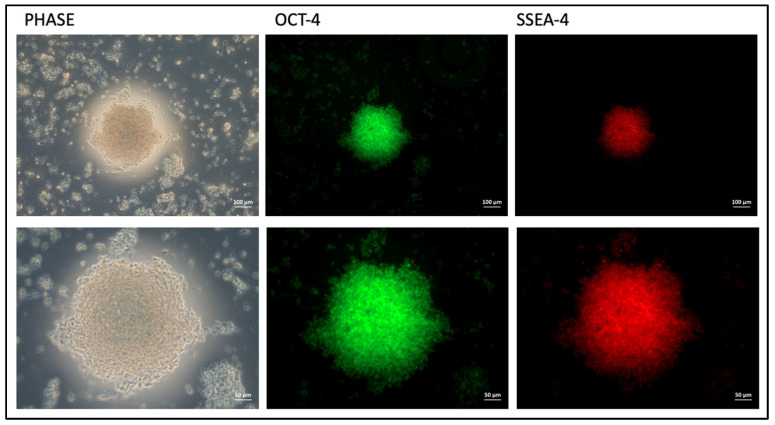
Immunostaining analysis results.

Under the inverted microscope, the immunostaining showed clear and distinct fluorescent signals associated with colony formation. In Figure 6, colony formation and distinct fluorescent emissions were clearly visible at 10× and 20× magnification. The green fluorescence from Alexa Fluor 488, indicating OCT-4 expression, and the red fluorescence from Alexa Fluor 568, marking SSEA-4 expression, clearly demonstrated the presence of pluripotent cells within the colonies. The strong and specific staining observed highlighted the effectiveness of the immunostaining protocol and the reliability of the chosen antibodies for iPSC characterization.

#### 3.4.3. Flow Cytometry Analysis Based on OCT-4 and SSEA-4 Antibody Expression

**Figure 7:**
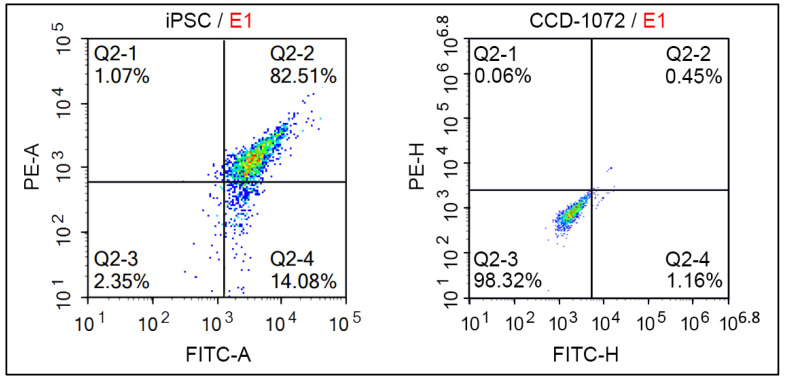
Flow cytometry analysis based on OCT-4 and SSEA-4 antibody expression.

OCT-4 is a key transcription factor essential for maintaining pluripotency in stem cells, whereas SSEA-4 is a surface marker frequently used to identify undifferentiated pluripotent cells [37]. The co-expression of these markers suggests a uniform iPSC population with a high potential for differentiation into various cell types. Flow cytometry analysis was used to characterize induced pluripotent stem cells (iPSCs) using OCT-4 and SSEA-4 antibodies tagged with FITC and PE fluorescent markers, respectively. Analysis included both a negative control and a gating strategy to ensure the accurate identification of the subpopulations. The initial gating was based on FSC-H vs. SSC-H to isolate the main target population, identified as the E1 Gate. Subsequent sub-gating was performed using FITC-A vs. PE-A within the E1 Gate, enabling the identification of specific subpopulations. For control, untransfected CCD-1072SK cells, which naturally lack the markers OCT-4 and SSEA-4, were used as negative controls to confirm the absence of non-specific staining. The results show that 82.51% of the cells co-expressed both FITC and PE markers, indicative of OCT-4 and SSEA-4 expression, as shown in Figure 7. In contrast, only 0.45% of the cells in the control group showed dual marker expression. The thorough methodology and inclusion of proper controls highlighted the robustness and reliability of the flow cytometry analysis.

#### 3.4.4. Analysis of Alkaline Phosphatase Activity

Alkaline phosphatase is an enzyme expressed in pluripotent cells and is commonly used as a marker to evaluate the maintenance of pluripotency in iPSC cultures. The alkaline phosphatase activity assay quantitatively measures ALP levels, confirming the undifferentiated states of iPSCs. In general, embryonic stem cells and iPSCs display high ALP activity, akin to what is seen in undifferentiated cells [38]. In contrast, differentiated and somatic cells typically exhibit lower ALP activity. As cells differentiate, they typically reduce ALP expression, making ALP activity a valuable marker for identifying stem cells in their undifferentiated state. ALP is a functional enzyme rather than a transcription factor or cell surface marker, making it an indirect indicator of pluripotency. Therefore, while ALP remains a useful tool for identifying pluripotent cells, it is often recommended to use it in conjunction with other more specific markers to accurately assess pluripotency.

**Figure 8:**
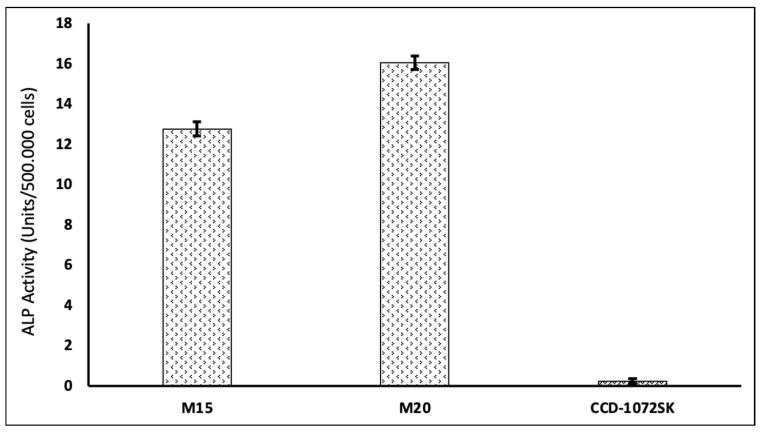
Relative ALP activity of untransfected cells (CCD1072-SK) and cells cultured on Matrigel (M15: day 15; M20: day 20; CCD1072-SK: control group).

The activity of alkaline phosphatase was assessed using the Elabscience ALP Activity Assay Kit (Cat. No.: E-BC-K091-M). The procedure recommended by the kit supplier was followed. The non-transfected CCD1072-SK cells and those cultured on Matrigel for 15 and 20 days post-transfection were compared against seven standard samples provided by the supplier (*n* = 3). The graph displaying the ALP activity (Unit/mL) values is shown in Figure 8.

After the experiment, CCD1072-SK, a somatic cell without gene transfer, showed an ALP activity of 0.23 units per 500,000 cells. Conversely, cells cultured in a specific medium on Matrigel for 15 days exhibited an ALP level of 12.77 units per 500,000 cells, which increased to 16.05 units per 500,000 cells after 20 days of culture.

#### 3.4.5. Quantitative Analysis of Gene Expression for Reprogramming Factors Using RT-qPCR

Real-time quantitative PCR (RT-qPCR) is a powerful and widely used technique for assessing gene expression levels, particularly in the characterization of induced pluripotent stem cells (iPSCs) [39,40]. By quantifying the expression of key pluripotency markers, RT-qPCR provides crucial insights into the reprogramming efficiency and status of iPSCs. In this study, RT-qPCR was employed to evaluate the expression levels of the core pluripotency factors OCT-4, SOX-2, and KLF-4, which are essential indicators of successful reprogramming [41].

**Figure 9:**
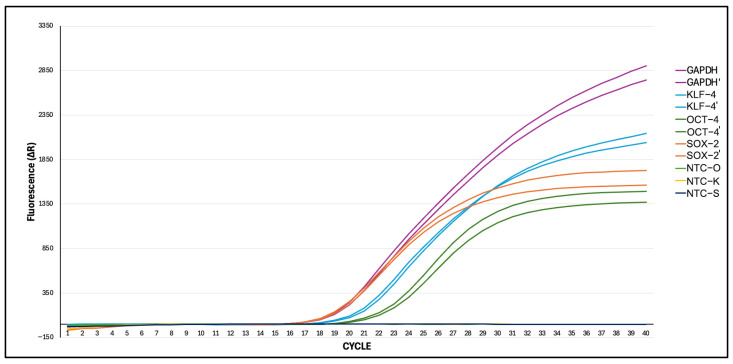
The amplification graph of SOX-2, OCT4, KLF-4 and control genes.

The results are illustrated in a color-coded amplification plot: the purple line represents the GAPDH gene, serving as the positive control reference gene, while the light green, yellow, and dark blue lines indicate the negative control non-transfected cells (NTC) for each gene. The amplification graph in Figure 9 shows an increase in gene expression, with OCT-4 expression detected after the 18th cycle (Ct: 18.4), SOX-2 after the 17th cycle (Ct: 17.2), and KLF-4 after the 19th cycle (Ct: 19.8). These results indicate that the mRNA levels of the reprogramming factors OCT-4, SOX-2, and KLF-4 are significantly higher in transfected cells compared to the NTC group, as confirmed by the RT-qPCR data.

### 3.5. Differentiation of iPSCs into Cardiomyocytes and Characterization

**Figure 10:**
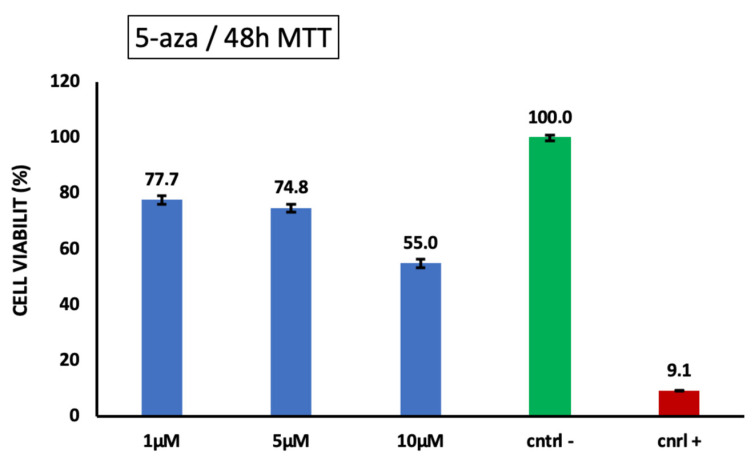
Cytotoxicity evaluation of iPSCs exposed to 5-Aza.

To assess the cytotoxic effects of 5-aza on iPSCs, MTT assays were conducted following a 2-day period of exposure to 5-aza. In summary, 10 cells were seeded into 96-well plates with 5 replicates and incubated overnight. The following day, the medium was replaced with a medium containing various concentrations of 5-aza (1 μM, 5 μM, and 10 μM), and the cells were cultured for an additional 2 days. After 48 h, the MTT assay was performed. DMSO was used as the positive control and m-TSER medium as the negative control. The OD was measured at 570 nm using a Thermo Scientific Multiskan Go ELISA reader. Cytotoxicity experiments were conducted in five replicates, and the mean value was used for further analysis. The results are shown in Figure 10. Based on the cell viability ratios, 5-aza demonstrated significant toxicity at a concentration of 10 μM, indicated by reduced cell viability. In contrast, concentrations of 1 µM and 5 µM resulted in cell survival rates of over 70%, suggesting minimal toxic effects at these lower concentrations.

**Figure 11:**
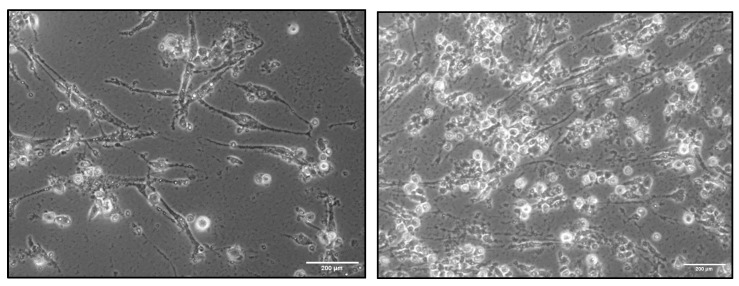
Morphological changes in iPSCs to cardiomyocytes-like cells.

Based on the MTT assay results, differentiation experiments on iPSCs were initiated using 1 μM and 5 μM concentrations of 5-aza. The iPSC colonies were cultured for two days with 1 μM and 5 μM of 5-aza. Subsequently, morphological changes were observed by culturing cells in standard medium for up to two weeks. During this period, significant morphological changes were observed in cells exposed to 5 μM concentrations, while no notable changes were seen in cells treated with 1 μM concentrations. The colonies treated with 5 μM began dispersing from the outer edge and exhibited a morphology characterized by elongated or rod-shaped cells. Figure 11 displays an image of cells resembling induced pluripotent stem cell-derived cardiomyocytes-like cells (iPSC-CMs) produced during the experiment.

**Figure 12:**
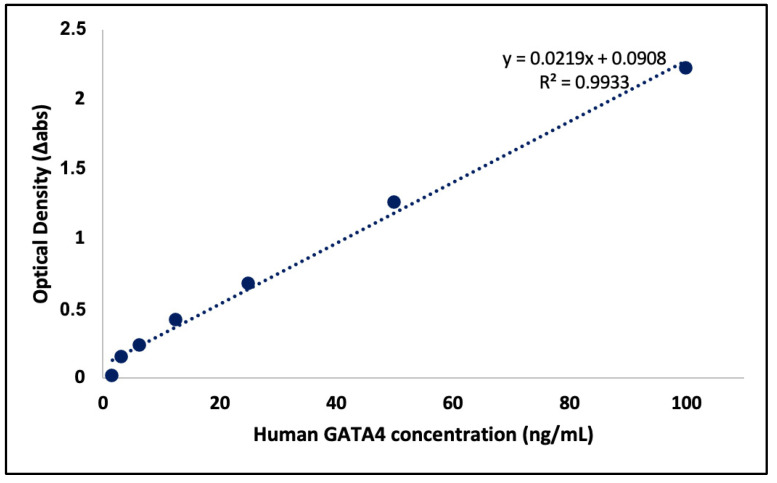
GATA-4 standart curve graph.

In addition to morphological changes, GATA-4 expression in the cells was assessed for more detailed characterization. The use of the Human GATA binding protein 4 kit allowed for the accurate measurement of GATA-4, a transcription factor essential for cardiac development. This offered valuable insights into the effectiveness and success of the differentiation process during the development of cardiomyocytes. The GATA4 ELISA kit was used to assess cardiomyocyte differentiation by analyzing morphological changes in the cells. Following the manufacturer’s recommended protocol, the kit yielded accurate results, which were calculated using a formula derived from the standard curve based on the measured absorbances. The healthy CCD1072 cell line was used as the control group, and all groups were studied in triplicate (*n* = 3). Standard curves are shown in Figure 12. The levels of GATA-4 in the experimental groups were determined using the formula provided in the graph.

The GATA-4 levels in CCD1072 cells, used as the control group, were below the detection limits of the ELISA kit, indicating negligible GATA-4 expression in these cells. In contrast, iPSC-CM cells exhibited a detectable GATA-4 level, measured at 9.79 ng/mL. These findings highlight the increased and specific expression of GATA-4 in iPSC-CM cells compared to control human fibroblast cells. The data obtained using the GATA-4 ELISA kit offered valuable insights into the successful differentiation of iPSCs into cardiomyocytes, highlighting the specificity of GATA-4 as a cardiac marker in the characterized cell population.

However, further characterization methods are necessary before we can conclusively state that the cells obtained are true cardiomyocytes. While the expression of GATA-4 is indicative of cardiac lineage, additional markers and functional assays must be employed to verify their identity as fully mature cardiomyocytes. Until then, we refer to these cells as cardiomyocyte-like cells, reflecting their potential and the early stages of differentiation. This cautious approach ensures that we accurately represent our findings and the current state of research.

## 4. Discussion

This study presents a new method in stem cell research, converting human fibroblast cells into induced pluripotent stem cells (iPSCs) with our specially designed solid lipid nanoparticles (OCTNPs), a non-viral delivery system. This method overcomes the limitations associated with viral vectors, paving the way for the use of iPSCs in regenerative medicine. The biocompatible non-viral vectors being used offer a promising approach for tissue repair. Additionally, using a non-viral carrier to deliver reprogramming factors helps to address concerns about the uncontrolled reprogramming of somatic cells to a totipotent state.

The transfection efficiency observed in our study was notably high compared to the literature, reaching 82.0% (±6.2). This was determined by counting the number of cells expressing GFP. This outperforms a variety of viral and non-viral gene delivery methods described in the current literature. Diecke, Sebastian et al. achieved the highest transfection efficiency, recorded at 21.45% ± 9.4%, using the COMİP 4in1 vector. The efficiency of the minicircle vector (7.6 kb) was similar to that of EBNA/OriP episomal plasmids (approximately 12 kb), with efficiencies of 15.05% ± 6.86% and 16.35% ± 5.87%, respectively [42]. In the study by Rezaee et al., the application of targeted liposomes as non-viral gene delivery systems achieved transfection rates ranging from 29.30% to 42.81% in the liver cells of mice [43]. Our approach not only shows greater effectiveness but also improves safety, a crucial aspect for advancing gene delivery technologies. This increased efficiency is especially significant in comparison to traditional viral vectors, known for their potential cytotoxic and immunogenic effects.

The presence of a GFP marker gene enables the more effective monitoring of morphological changes and colony formation in Matrigel due to reprogramming, as well as the precise control of transfection. A noticeable trend in colonization was observed starting from day 10. This observation is supported by evidence found in the literature. Swaidan, Nuha T. et al., found that specific mutations linked to human diseases impact the efficiency of reprogramming. The reprogramming results showed that iPSC colonies started to appear as early as day 10, with most colonies being visible by day 18 [44]. Ghasemi-Dehkordi, Payam et al. conducted a study in which they dissociated transduced human dermal fibroblasts six days after transduction. The reprogrammed cells were then cultured in hiPS medium on BD Matrigel to generate hiPSC colonies. The morphology of hiPSCs was observed from day 9 to day 14 [45]. The colonization tendencies and cell images observed in our study align with similar results reported in the literature. Studies by Maherali, Nimet and Konrad Hochedlinger (2008) [46], as well as Lizier, Nelson F., Irina Kerkis, and Cristiane V. Wenceslau (2013) [47], demonstrated iPSC morphologies that closely resembled those found in our results, further supporting our findings. The detailed chemical characterization of the derived iPSCs, conducted through immunofluorescence staining, ALP assay, and flow cytometry, confirmed their pluripotent state. The expression profiles of pluripotency markers (OCT-4 and SSEA-4), as measured by flow cytometry, and the observed staining patterns for pluripotency markers, such as OCT-4 and SOX-2, aligned closely with established data in the Skamagki, Maria et al. (2017), providing robust evidence for the successful reprogramming of the cells into iPSCs [48]. All data obtained confirmed that CCD1072-SK cells were successfully reprogrammed into iPSCs by the end of the culture period. Previous research employed small molecules to facilitate the reprogramming and differentiation of somatic cells by targeting specific signaling pathways and altering the epigenome [49,50]. Our approach integrates these insights with an innovative delivery method, improving both efficacy and safety.

This study is the first to report the reprogramming of human fibroblast cells into iPSCs using octadecylamine-based solid lipid nanoparticles, followed by their characterization and successful differentiation into cardiomyocyte-like cells using 5-azacytidine. Another unique aspect is the differentiation of iPSCs into cardiomyocyte-like cells for the first time using 5-azacytidine, paving the way for efficient and safe iPSC generation and cardiac lineage commitment. During the 15-day differentiation period, the iPSCs exhibited morphological changes such as dispersed colonies and elongated or rod-shaped cells, characteristics typical of induced pluripotent stem cell-derived cardiomyocyte-like cells (iPSC-CMs). GATA-4 expression further confirmed successful differentiation into cardiomyocytes. The use of 5-azacytidine in iPSC-CM differentiation highlights the potential clinical applications of this method.

## 5. Conclusions

In conclusion, this research not only introduces a novel technique for cellular reprogramming but also provides a comprehensive framework for characterization. The importance of this study goes beyond iPSC technology, setting the stage for progress in cardiac tissue engineering and particularly in nanobiotechnology. The findings of this study are highly promising for the field of regenerative medicine, as they address critical issues related to viral vectors and open up new possibilities for clinical applications.

## Data Availability

The datasets and materials used or analyzed during the current study are available from the corresponding author upon reasonable request.
